# Supplementary material for: Host interactors of effector proteins of the lettuce downy mildew Bremia lactucae obtained by yeast two-hybrid screening
Source: PLoS One. 2020 May 12;15(5):e0226540. doi: 10.1371/journal.pone.0226540 (PMC7217486; doi:10.1371/journal.pone.0226540)
Supplement: S1 Table — (DOCX) [file pone.0226540.s001.docx]

**S1 Table, Primers used in this study.**

| **Primers for amplification of lettuce gene fragments in Y2H vectors** | | |
| --- | --- | --- |
| *Name* | *Orientation* | *Sequence* |
| pDEST22 | Fwd | TATAACGCGTTTGGAATCACT |
| AP22 | Rev | GCTTTCGTGTGATGAACCCA |
| pDEST22/32 | Rev | AGCCGACAACCTTGATTGGAGAC |

| **Primers with BP cloning sites for full-length gene amplification** | | |
| --- | --- | --- |
| *Gene* | *Orientation* | *Sequence^1^* |
| *LsRTNLB05* | Fwd | *GGGGACAAGTTTGTACAAAAAAGCAGGCTTC*ATGTCGTTATGGCGCCAGC |
| *LsRTNLB05* | Rev | *GGGGACCACTTTGTACAAGAAAGCTGGGTT*TTATGCAAACTTCTTCGCTTTTG |
| *LsERF093* | Fwd | *GGGGACAAGTTTGTACAAAAAAGCAGGCTTC*ATGTGTGGTGGTGCGATCAT |
| *LsERF093* | Rev | *GGGGACCACTTTGTACAAGAAAGCTGGGTT*TTAGAAAGATCCATCCATAATGA |
| *LsFLX-like2* | Fwd | *GGGGACAAGTTTGTACAAAAAAGCAGGCTTC*ATGGGAAGCAAAGGTAGACTT |
| *LsFLX-like2* | Rev | *GGGGACCACTTTGTACAAGAAAGCTGGGTT*CTATCTCCGGGCAGGGTG |
| *LsHSP90-11* | Fwd | *GGGGACAAGTTTGTACAAAAAAGCAGGCTTC*ATGGGGGACACAGAGACGT |
| *LsHSP90-11* | Rev | *GGGGACCACTTTGTACAAGAAAGCTGGGTT*TTAGTCGACCTCTTCCATCTT |
| *Lsa008464.1* | Fwd | *GGGGACAAGTTTGTACAAAAAAGCAGGCTTC*ATGGCGTCTACGACCACCG |
| *Lsa008464.1* | Rev | *GGGGACCACTTTGTACAAGAAAGCTGGGTT*CTATTTCATTGCATCGACTTCA |
| *LsDjA2* | Fwd | *GGGGACAAGTTTGTACAAAAAAGCAGGCTTC*ATGTTTGGAAGACAGCCGAAG |
| *LsDjA2* | Rev | *GGGGACCACTTTGTACAAGAAAGCTGGGTT*TCATTGCTGCGCACATTGCA |
| *LsCSN5* | Fwd | *GGGGACAAGTTTGTACAAAAAAGCAGGCTTC*ATGGATCCCTACTCCTTCTC |
| *LsCSN5* | Rev | *GGGGACCACTTTGTACAAGAAAGCTGGGTT*TCAAGATTGAACCATAGGCTC |
| *Lsa015570.1* | Fwd | *GGGGACAAGTTTGTACAAAAAAGCAGGCTTC*ATGGCTGTGATGAACGGTGG |
| *Lsa015570.1* | Rev | *GGGGACCACTTTGTACAAGAAAGCTGGGTT*CTAGTTGACTACAACCGGTG |
| *Lsa021294.1* | Fwd | *GGGGACAAGTTTGTACAAAAAAGCAGGCTTC*ATGTTGGCGAAAGAATCGCTT |
| *Lsa021294.1* | Rev | *GGGGACCACTTTGTACAAGAAAGCTGGGTT*TCAATGGAAGTTACGAGTGATT |
| *LsBPM3* | Fwd | *GGGGACAAGTTTGTACAAAAAAGCAGGCTTC*ATGCTTGTCAATAATTTCGATCA |
| *LsBPM3* | Rev | *GGGGACCACTTTGTACAAGAAAGCTGGGTT*TTACAACCGTCTACGCATACG |
| *LsFER3* | Fwd | *GGGGACAAGTTTGTACAAAAAAGCAGGCTTC*ATGTCGATGAACACAGCCATT |
| *LsFER3* | Rev | *GGGGACCACTTTGTACAAGAAAGCTGGGTT*TCAAACACCCTCCTCAAGAAG |
| *LsNAC069* | Fwd | *GGGGACAAGTTTGTACAAAAAAGCAGGCTTC*ATGGGTTCTGACTTGATTGAAG |
| *LsNAC069* | Rev | *GGGGACCACTTTGTACAAGAAAGCTGGGTT*TTACCATATACACCTACCCAAT |

*^1^* Sequences in italic represent attB cloning sites.
