# Supplementary material for: Host interactors of effector proteins of the lettuce downy mildew Bremia lactucae obtained by yeast two-hybrid screening
Source: PLoS One. 2020 May 12;15(5):e0226540. doi: 10.1371/journal.pone.0226540 (PMC7217486; doi:10.1371/journal.pone.0226540)
Supplement: S2 Table — (DOCX) [file pone.0226540.s002.docx]

**S2 Table, Prediction of importin-α-dependent nuclear localization signals^1^**

| *Gene* | *NLS start position^2^* | *NLS sequence* | *Score^3^* |
| --- | --- | --- | --- |
| BLG02 | 95 | KQTKRKRPKD | 10 |
| BLG03 | 12 | RPQGKLRVNAATNVESDERFLDGLKALVRGFY | 3.3 |
| BLN01 | - | - | - |
| BLN03 | 3 | QLKRLSVTYGSAFLFVIGFIVLLAFAMTA | 3 |
| BLN04 | 22 | RLKGSSKVSKATTGSEERFIRSQLQFLINKIFG | 2.8 |
| BLR05 |  | - |  |
| BLR08 | 70 | ESKHELPSIMSFIGPAAAGVLAIVLIGAVI | 2.2 |
| BLR09 | 14 | ENRRLRPRVEPTANELDKQSDVDTKLEAD | 3.2 |
| BLR11 | 390 | KTAKKRGTFDVVTPEAKKINH | 4.5 |
| BLR12 | 22 | RIARRYLRETAAMEGQELEKNLGFDQTIKNVKKP | 5.2 |
| BLR18 | - | - | - |
| BLR20 | - | - | - |
| BLR27 | 13 | LGKSRQLRSSVELVEGLLLTSDKLAKVI | 3 |
| BLR28 | 220 | RFVLDNFKECRATIRYGTVEDWYKHPMLNKLLRV | 4.3 |
| BLR38 | 136 | MPSSRKRPRALDE | 9.5 |
| LsRTNLB05 | 269 | FGKVQNHYSKLDTSLLSRIPKAKAKKF | 3.9 |
| LsERF093 | 100 | SSRKRKNQYR | 8 |
| LsFLX-like2 | 6 | RLPPPHHLRRPLPGPGIGHHDSIPPEIHPQHGRFPP | 4.3 |
| LsHSP90-11 | - | - | - |
| Lsa008464.1 | 142 | RRLDFGSDSDEEEYDVDDEDDVTHNKNGY | 2.9 |
| LsDjA2 | 265 | PKFKRKGDDL | 5 |
| LsCSN5 | 57 | DPHYFKRVKVS | 5 |
| Lsa015570.1 | 216 | GVSKSASFAKRSLSVDTLHNTPRNYKEPAP | 3.2 |
| Lsa021294.1 | 281 | FRQVRSRLSYEQFAAFLANVKELNSQKIYIYI | 3.9 |
| LsBPM3 | - | - | - |
| LsFER3 | 11 | SRVLLKKPDVDLDSVPKNTIGSVKIPCFS | 3.7 |
| LsNAC069 | 21 | GKCFPPGFRFHPTDEELVLYYLKRKIC | 4.3 |

^1^ Predicted using cNLS mapper

^2^ If multiple NLSs were detected, the highest scoring NLS is given

^3^ Higher scores are associated with a stronger NLS activity and more frequent nuclear localization (i.e. a score of >8 indicates exclusively nuclear). A cut-off of 4 was used.
