# Supplementary material for: Host interactors of effector proteins of the lettuce downy mildew Bremia lactucae obtained by yeast two-hybrid screening
Source: PLoS One. 2020 May 12;15(5):e0226540. doi: 10.1371/journal.pone.0226540 (PMC7217486; doi:10.1371/journal.pone.0226540)
Supplement: S3 Table — (DOCX) [file pone.0226540.s003.docx]

|  |  | | | | | |
| --- | --- | --- | --- | --- | --- | --- |
| *Effector* | *TOPCONS* | *OCTOPUS* | *Philius* | *PolyPhobius* | *SCAMPI* | *SPOCTOPUS* |
| BLN03 | 148-168 | 148-168 | 145-167 | 145-167 | 5-25; 148-168 | 148-168 |
| BLN04 | 117-137 | - | 116-137 | 115-137 | - | 115-135 |
| BLR05 | - | - | 51-73 | 51-71 | - | 53-73 |
| BLR08 | 81-101 | - | 78-101 | 78-101 | - | 80-100 |
| BLR09 | 70-90 | - | 69-90 | 69-90 | - | 68-88 |
| BLR12 | 79-99 | - | 81-98 | 81-98 | - | 79-99 |

**S3 Table, Position of transmembrane domains in effectors using TOPCONS^1^.**

**^1^** TOPCONS analyses sequences with the individual topology prediction methods OCTOPUS, Philius, PolyPhobius, SCAMPI and SPOCTOPUS and provides both the individual output as well as a consensus prediction
