# Supplementary material for: Host interactors of effector proteins of the lettuce downy mildew Bremia lactucae obtained by yeast two-hybrid screening
Source: PLoS One. 2020 May 12;15(5):e0226540. doi: 10.1371/journal.pone.0226540 (PMC7217486; doi:10.1371/journal.pone.0226540)
Supplement: S4 Table — (DOCX) [file pone.0226540.s004.docx]

| Effector | Position | Peptide | Score | Cutoff^2^ | Modification type |
| --- | --- | --- | --- | --- | --- |
| BLR12 | 98 | PFGIFYVCNR | 6.131 | 4.003 | S-Farnesylation: Non-consensus |
| BLR12 | 98 | PFGIFYVCNR | 7.422 | 1.617 | S-Geranylgeranylation: Non-consensus |
| BLR09 | 87 | GVSIISACYGIT | 14.294 | 4.003 | S-Farnesylation: Non-consensus |
| BLR05 | 72 | FAIMALRCLPFCL | 7.46 | 6.806 | S-Farnesylation: CAAX |
| BLG03 | 9 | SSVTTEEGDGRPQGK | 9.32 | 9.218 | N-Myristoylation: Non-consensus |

**S4 Table, Putative post-translational lipid modifications** **in membrane-associated effectors^1^.**

^1^ Predicted using GPS-Lipid/CSS-Palm

^2^ The chosen threshold is high
