# Supplementary material for: Host interactors of effector proteins of the lettuce downy mildew Bremia lactucae obtained by yeast two-hybrid screening
Source: PLoS One. 2020 May 12;15(5):e0226540. doi: 10.1371/journal.pone.0226540 (PMC7217486; doi:10.1371/journal.pone.0226540)
Supplement: S1 File — (DOC) [file pone.0226540.s005.doc]

**Library screening**

The mating method was used for library screening [1]. A 1 ml yeast library aliquot was thawed on ice and used to inoculate 100 ml YEPD medium. After incubation with shaking at 28 °C for 1 hour, the yeast cells were spun down at 1800 rpm for 5 min, washed twice with ddH_2_O and resuspended to an OD_600_ of 1 in YEPD medium. 6 ml of yeast library was mixed with an equal amount of overnight cultured bait strain cells and yeast cells were collected by centrifugation. The pellet was resuspended in 300 µl ddH_2_O and plated on YEPD medium + 100 μg/ml ampicillin. After incubation at 30 °C for 4 hours, 2 ml ddH_2_O was added to the plate and yeast was scraped off. Yeast was collected by centrifugation and the pellet was resuspended in 600 µl ddH_2_O. Yeast was plated on Sc –Leu –Trp –His + amp medium and incubated at 30 °C for four days. A 1:10,000 dilution was plated on Sc –Leu –Trp + amp medium to determine the number of diploid yeast cells screened. Per bait a minimum of one million diploid yeast cells were screened.

Up to 96 colonies per Sc –Leu –Trp –His + amp plate were picked, resuspended in 25 μl ddH_2_O and spotted on fresh Sc –Leu –Trp –His + amp medium in duplo. After incubation at 30 °C for two days, one plate was used for replica plating and the other for colony PCR. Yeast was replica plated on Sc –Leu –Trp –His + 2 mM 3-amino-1,2,4-triazole (3AT) (Formedium) + amp medium and Sc –Leu –Trp –His +5 mM 3AT + amp medium followed by incubation at 30 °C for two days, and plated on Sc –Leu –Trp –Ade + amp medium followed by incubation at 20 °C for five days.

For yeast colony PCR, yeast patches from a Sc –Leu –Trp –His + amp plate were lightly touched with a pipette tip, resuspended in 30 μl 0.02 M NaOH and heated at 99 °C for 10 min. Lysates were spun down briefly and 1 μl of the supernatant was used for a 10 μl PCR reaction with DreamTaq DNA polymerase (ThermoScientific) and primers pDEST22 and AP22 (S1 Table) to detect a DNA fragment that recurred as unspecific ‘interactor’ with almost all bait screened. The prey fragment from colonies negative for the unspecific ‘interactor’ was amplified with primers pDEST22 and pDEST22/32 (S1 Table). Prey PCR products were purified using Agencourt AMPure XP beads according to the manufacturer’s protocol and Sanger sequenced. Prey sequences were used in a BlastN search against a previously described lettuce transcriptome [2] to identify the corresponding scaffold/ contig on which the lettuce cDNA fragment was located. A representative colony per identified scaffold/ contig was grown o/n in 4 ml YEPD and 1-3 ml was used for plasmid isolation. Yeast was spun down at 2000 rpm for two minutes and the pellet was resuspended in 1 ml of TE (10 mM Tris-HCl, 1 mM EDTA, pH 7.5). The centrifugation step was repeated, and the pellet was resuspended in 200 µl Resuspension Buffer with RNase A supplemented with 15 U Zymolyase-20T (Amsbio #120491-1) and 5 µl 2-mercaptoethanol. After incubation at 37 °C for 60 minutes, plasmid DNA was purified using the GenElute Plasmid miniprep kit (Sigma). Due to the low plasmid DNA yield of yeast, prey plasmid was transformed in *E. coli* DH5α cells and plasmid DNA isolated from a single *E. coli* colony was used for sequencing and retransformation.

To confirm the interaction found in the library screen, bait and prey plasmid were cotransformed in yeast strain Y8930, selected on Sc –Leu –Trp + amp medium and replica plated on Sc –Leu –Trp –His + amp, Sc –Leu –Trp –His + 2 mM 3AT + amp, Sc –Leu –Trp –His + 5 mM 3AT + amp and Sc –Leu –Trp –Ade + amp medium.

**Gene nomenclature**

- Gene models containing domains PF00226 (DNAJ domain), PF01556 (DNAJ C terminal domain) and PF00684 (cysteine containing DNAJ central domain) were named *LsDjA* according to nomenclature proposed by Rajan and D’Silva [3].
- Gene models containing domain PF00210 (Ferritin) were named *LsFER* conform nomenclature used by by Petit and coworkers [4].
- Gene models containing domain PF00183 (HSP90) were named *LsHSP90* according to nomenclature proposed by Krishna and Gloor [5].
- Gene models containing domain PF02453 (Reticulon) were named *LsRTLNB* according to nomenclature proposed by Oertle and coworkers [6].
- Gene models containing domain PF01398 (JAB1/Mov34/MPN/PAD-1 ubiquitin protease) were further analysed by BLASTp. Only gene model Lsat_1_v5_gn_3_9100.1 (Lsa011822.1) displayed homology to CSN5 and was therefore named *LsCSN5*.
- Gene models containing domain PF03208 (PRA1) were translated and aligned with Arabidopsis PRA1 proteins using Clustal Omega [7] with default parameters. A phylogenetic tree was constructed using MEGA 7.0 [8] using the Neighbor-Joining method with partial deletion of sites with less than 95% coverage, and bootstrap values based on 1000 iterations. The tree was visualized using iTOL software [9] and used to define clades for lettuce PRA1 proteins. Lettuce PRA1 gene models were named accordingly [10].
- Gene models containing domain PF00651 (BTB/POZ domain) were further analysed using HMMSCAN for the presence of other (statistically non-significant) domains. Due to the presence of a not significant MATH domain in LsBPM3 (Lsa022944.1), all BTB/POZ domain containing lettuce proteins were aligned with AtBPM1-AtBPM6 using Clustal Omega [7] with default parameters. A phylogenetic tree was constructed using MEGA 7.0 [8] using the Neighbor-Joining method with pairwise deletion to accommodate the presence of diverse domains, and bootstrap values based on 1000 iterations. The alignment was manually inspected and poorly aligned sequences were removed. Visualization of the resulting tree (Fig S3) showed that five lettuce proteins grouped with the six AtBPM proteins including LsBPM3. The five lettuce proteins were named according to nomenclature proposed by Weber and coworkers [11].
- Gene models containing domain PF00847 (AP2 domain) were further analysed for the presence of domain PF02362 (B3 domain). Three proteins contained an AP2 and B3 domain and thereby constitute the RAV family in lettuce. All AP2 domain containing lettuce proteins were aligned using Clustal Omega [7] with default parameters. A phylogenetic tree was constructed using MEGA 7.0 [8] using the Neighbor-Joining method with pairwise deletion to accommodate the presence of diverse domains, and bootstrap values based on 1000 iterations. The alignment was manually inspected and poorly aligned sequences were removed. 22 AP2 domain containing proteins constituted a separate clade. In 20 of these, two AP2 domains had been found. HMMSCAN analysis of the other two proteins revealed that these contained a second non-significant AP2 domain. Due to the clear grouping, all 22 proteins were considered members of the AP2 family. The remaining 185 lettuce gene models were named *LsERF*.
- Due to the absence of significant domains in Lsa007018.1 (gene model Lsat_1_v5_gn_2_86420.1), a BLASTp search was performed to identify homologous proteins in Arabidopsis. The best hits contained member of the FLX-like family in Arabidopsis. A tBLASTn search was performed at CoGe using FLX (At2g30120.2), FLX-like1 (At3g14750.1), FLX-like2 (At1g67170.1), FLX-like3 (At1g55170.1) and FLX-like4 (AT5G61920.1) as query sequences against the *Lactucae sativa* (ID35223) v8 genome with an E-value cut-off of 1e-5. The ten identified lettuce sequences, including gene model Lsat_1_v5_gn_2_86420.1, were aligned with the Arabidopsis sequences using Clustal Omega [7] with default parameters. A phylogenetic tree was constructed using MEGA 7.0 [8] using the Neighbor-Joining method with partial deletion of sites with less than 90% coverage, and bootstrap values based on 1000 iterations. The tree (Fig S4) was visualized using iTOL software [9] and used to define the orthologs of AtFLX. The remaining eight gene models were named *LsFLX-like* conform nomenclature used by Choi and coworkers [12].
- Three targets - Lsa008464.1, Lsa015570.1 and Lsa021294.1 - on the short-list neither contained Pfam domains with a significant *E*-value nor did they display sequence homology to previously named genes in Arabidopsis.

**References**

1. Fromont-Racine M, Rain JC, Legrain P. Building protein-protein networks by two-hybrid mating strategy. Methods Enzymol. 2002;350: 513–524. doi:10.1016/S0076-6879(02)50982-4

2. Pelgrom AJE, Eikelhof J, Elberse J, Meisrimler C-N, Raedts R, Klein J, et al. Recognition of lettuce downy mildew effector BLR38 in *Lactuca serriola* LS102 requires two unlinked loci. Mol Plant Pathol. 2018; 20: 240-253. doi:10.1111/mpp.12751

3. Rajan VB V, D’Silva P. *Arabidopsis thaliana* J-class heat shock proteins: Cellular stress sensors. Funct Integr Genomics. 2009;9: 433–446. doi:10.1007/s10142-009-0132-0

4. Petit JM, Briat J-F, Lobréaux S. Structure and differential expression of the four members of the *Arabidopsis thaliana* ferritin gene family. Biochem J. 2001;359: 575–582. doi:10.1042/0264-6021:3590575

5. Krishna P, Gloor G. The Hsp90 family of proteins in *Arabidopsis thaliana*. Cell Stress Chaperones. 2001;6: 238–246. doi:10.1379/1466-1268(2001)006<0238:THFOPI>2.0.CO;2

6. Oertle T, Klinger M, Stuermer CAO, Schwab ME. A reticular rhapsody: Phylogenic evolution and nomenclature of the RTN/Nogo gene family. FASEB J. 2003;17: 1238–1247. doi:10.1096/fj.02-1166hyp

7. Sievers F, Wilm A, Dineen D, Gibson TJ, Karplus K, Li W, et al. Fast, scalable generation of high-quality protein multiple sequence alignments using Clustal Omega. Mol Syst Biol. 2014;7: 539–539. doi:10.1038/msb.2011.75

8. Kumar S, Stecher G, Tamura K. MEGA7: Molecular evolutionary genetics analysis version 7.0 for bigger datasets. Mol Biol Evol. 2016;33: 1870–1874. doi:10.1093/molbev/msw054

9. Letunic I, Bork P. Interactive tree of life (iTOL) v3: An online tool for the display and annotation of phylogenetic and other trees. Nucleic Acids Res. 2016;44: W242–W245. doi:10.1093/nar/gkw290

10. Alvim Kamei CL, Boruc J, Vandepoele K, Van den Daele H, Maes S, Russinova E, et al. The *PRA1* gene family in Arabidopsis. Plant Physiol. 2008;147: 1735–1749. doi:pp.108.122226 [pii]\r10.1104/pp.108.122226

11. Weber H, Bernhardt A, Dieterle M, Hano P, Mutlu A, Estelle M, et al. Arabidopsis AtCUL3a and AtCUL3b Form Complexes with Members of the BTB/POZ-MATH Protein Family. Plant Physiol. 2005;137: 83–93. doi:10.1104/pp.104.052654

12. Choi K, Kim J, Hwang H, Kim S, Park C, Kim SY, et al. The FRIGIDA complex activates transcription of *FLC*, a strong flowering repressor in *Arabidopsis*, by recruiting chromatin modification factors. Plant Cell. 2011;23: 289–303. doi:10.1105/tpc.110.075911
