# Supplementary figures and images for: Host interactors of effector proteins of the lettuce downy mildew Bremia lactucae obtained by yeast two-hybrid screening
Source: PLoS One. 2020 May 12;15(5):e0226540. doi: 10.1371/journal.pone.0226540 (PMC7217486; doi:10.1371/journal.pone.0226540)

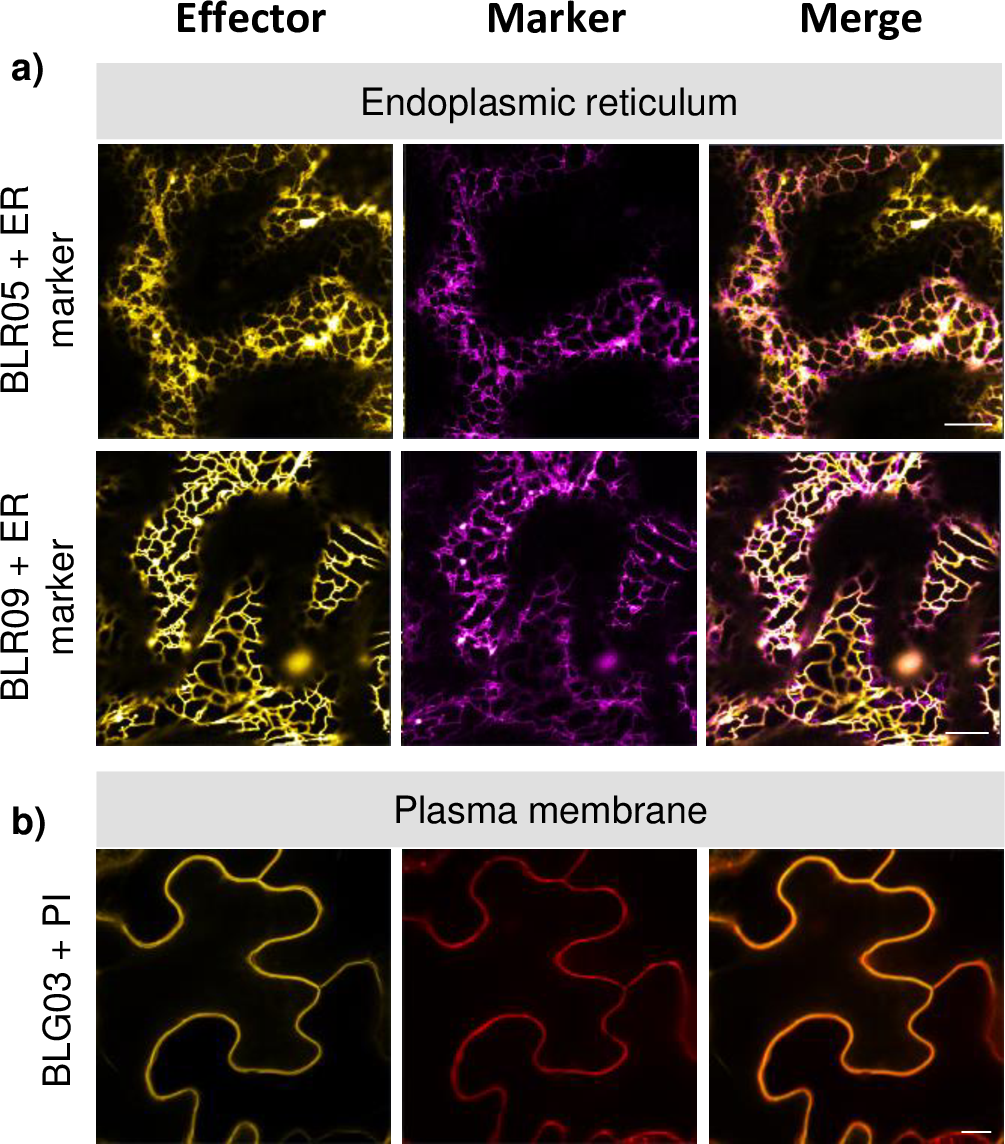

Supplement: S1 Fig — A) YFP-BLR05 and YFP-BLR09 co-localize with an CFP-tagged ER lumenal marker. B) YFP-BLG03 localizes to the plant plasma membrane. Leaf sections were incubated in propidium iodide (PI) to stain the cell wall. Bars = 10 μm. (TIF) [file pone.0226540.s007.tif]

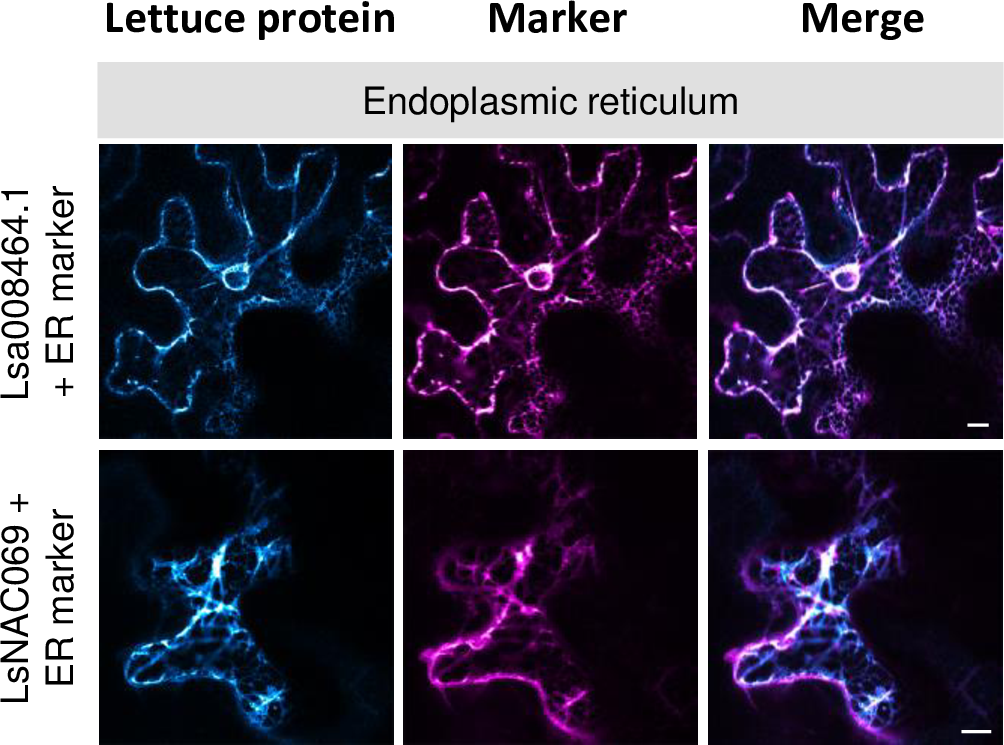

Supplement: S2 Fig — Bars = 10 μm. (TIF) [file pone.0226540.s008.tif]

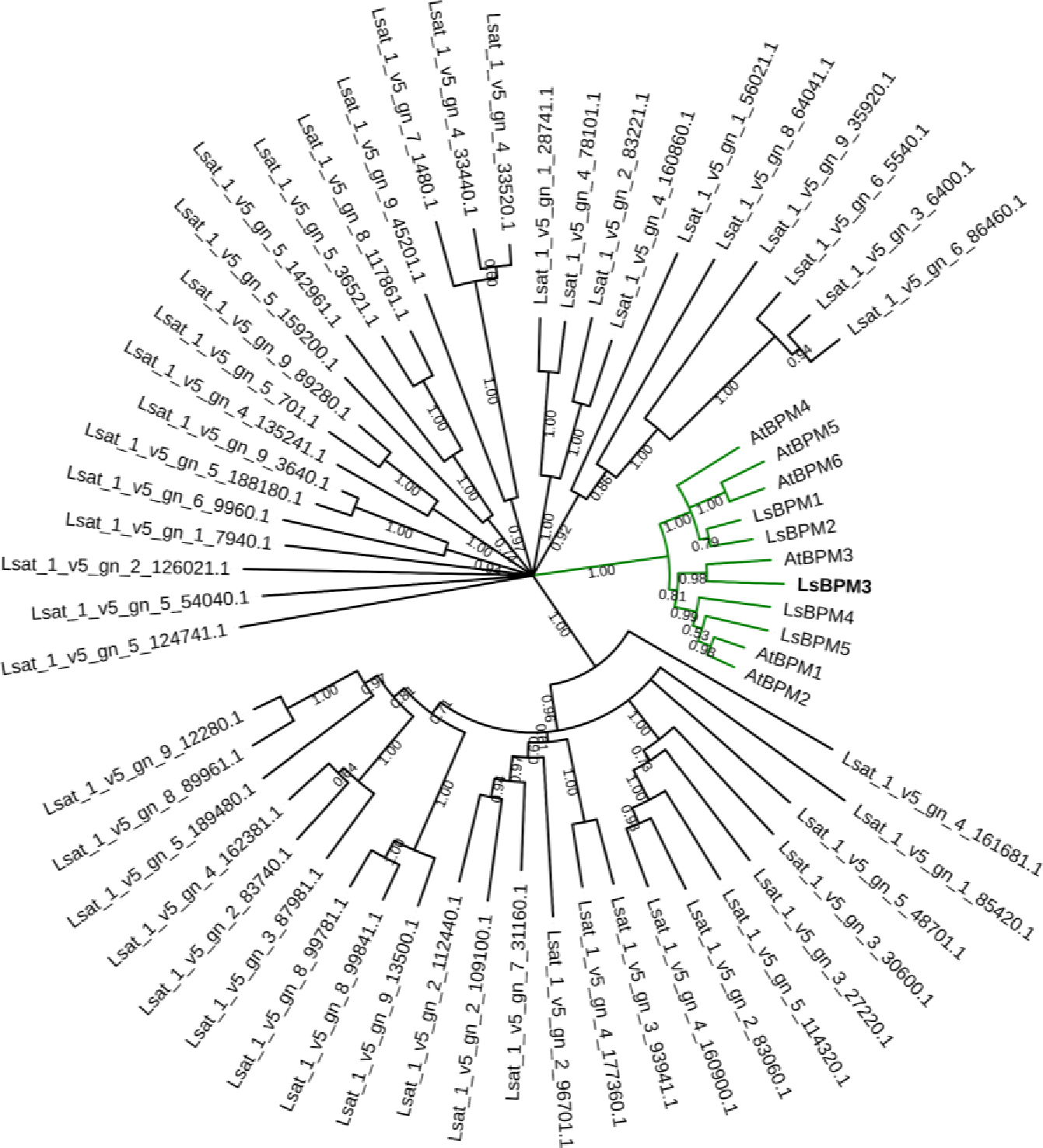

Supplement: S3 Fig — The Y2H identified target, LsBPM3, forms a distinct clade with four other lettuce BTB/POZ domain containing proteins and six Arabidopsis BPM proteins. AtBPM1-AtBPM6 contain in addition to a BTB/POZ domain also a MATH domain. Protein sequences were aligned using Clustal Omega, a Neighbor-Joining phylogenetic tree was constructed using MEGA7 and the tree was visualized using iTOL software. (TIF) [file pone.0226540.s009.tif]

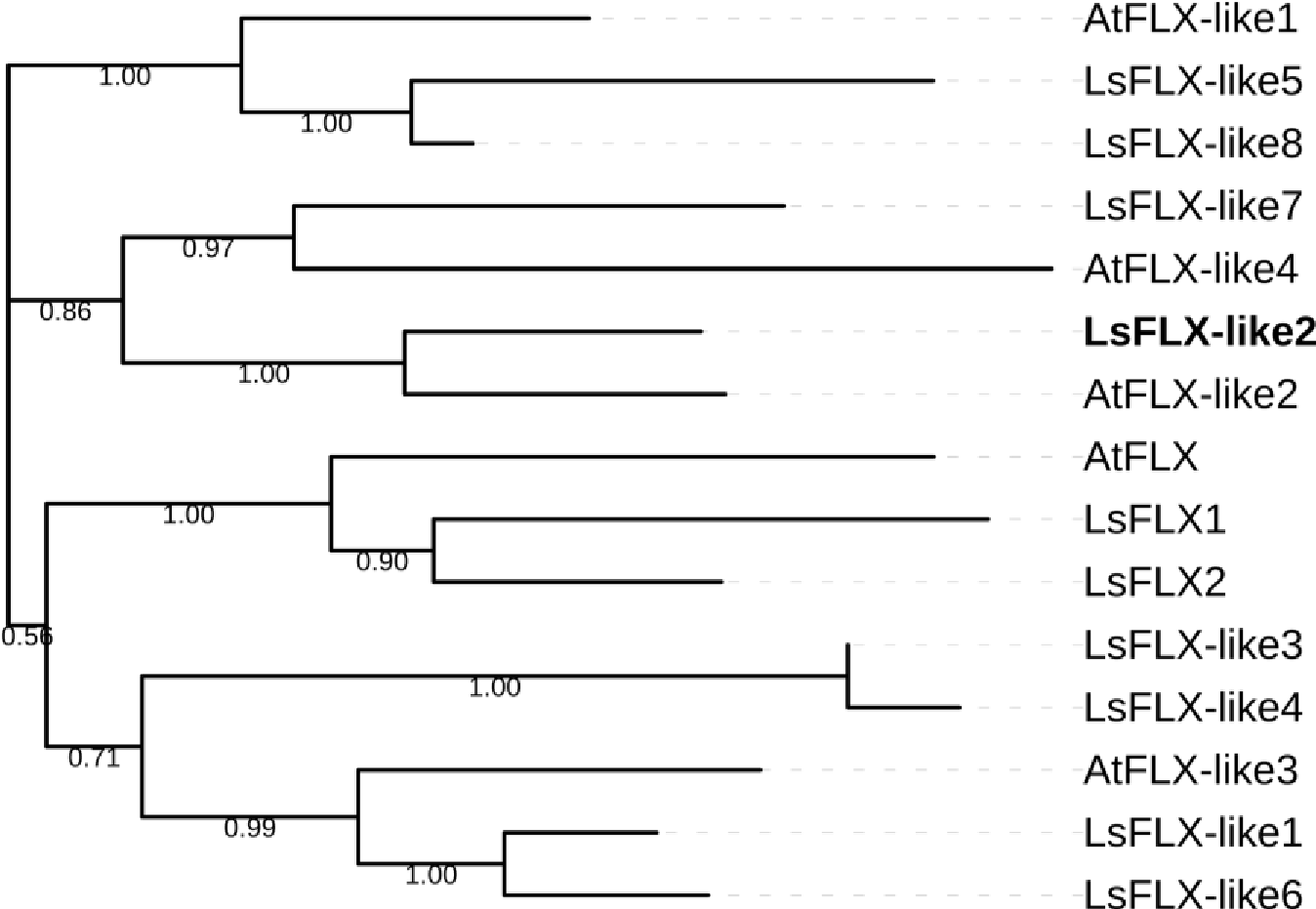

Supplement: S4 Fig — Protein sequences were aligned using Clustal Omega, a Neighbor-Joining phylogenetic tree was constructed using MEGA7 and the tree was visualized using iTOL software. (TIF) [file pone.0226540.s010.tif]
