# Supplementary material for: Host interactors of effector proteins of the lettuce downy mildew Bremia lactucae obtained by yeast two-hybrid screening
Source: PLoS One. 2020 May 12;15(5):e0226540. doi: 10.1371/journal.pone.0226540 (PMC7217486; doi:10.1371/journal.pone.0226540)
Supplement: S1 Raw images — (PDF) [file pone.0226540.s011.pdf]

Chemiluminescence _blot _effector proteins1

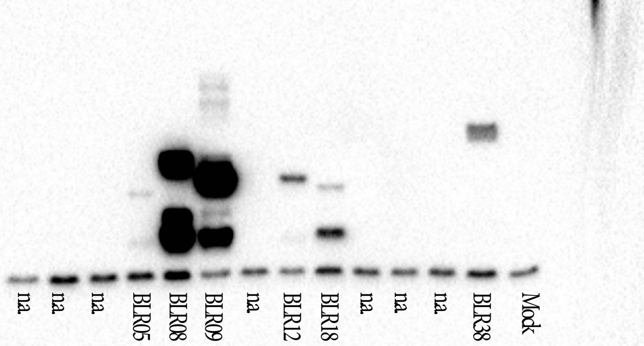

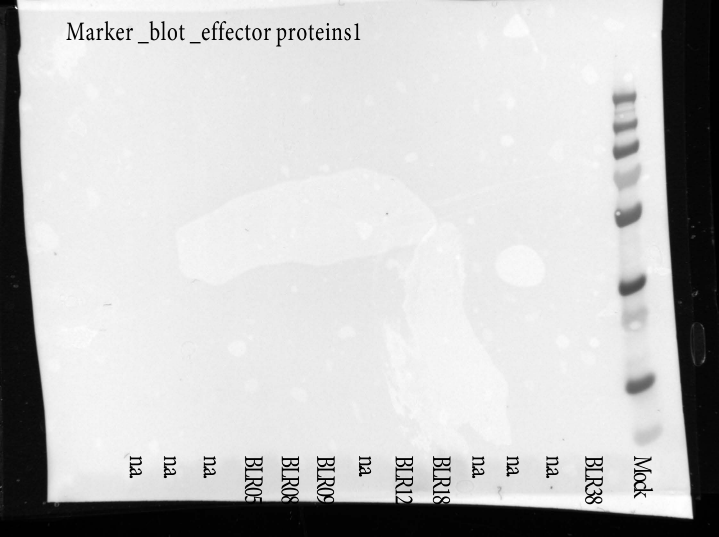
Marker _blot _effector proteins1
n.a.
n.a.
n.a.
BLR05
BLR08
BLR09
n.a.
BLR12
BLR18
n.a.
n.a.
n.a.
BLR38
Mock

Chemiluminescence _blot _target proteins1

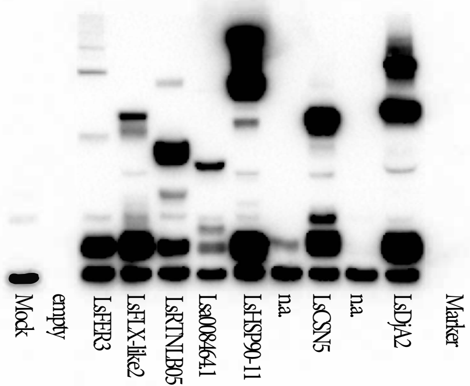

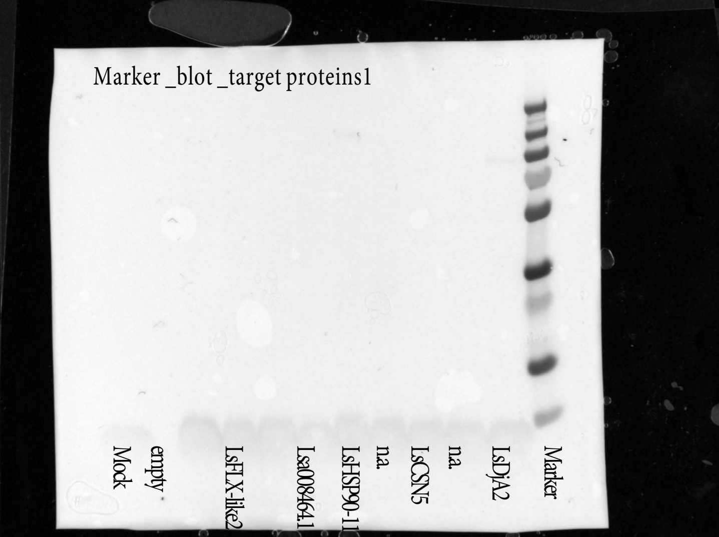

Marker _blot _target proteins1
Mock
empty
LsFLX-like2
Lsa008464.1
LsHSP90-1
n.a.
LsCSN5
n.a.
LsDjA2
Marker

Chemiluminescence_blot_target_proteins2 and BLG02

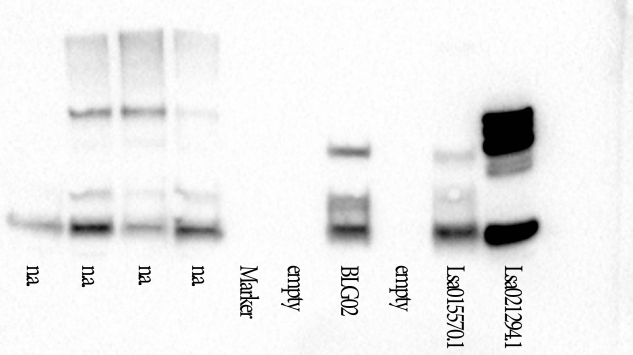

Marker_blot _target_proteins2 and BLG02

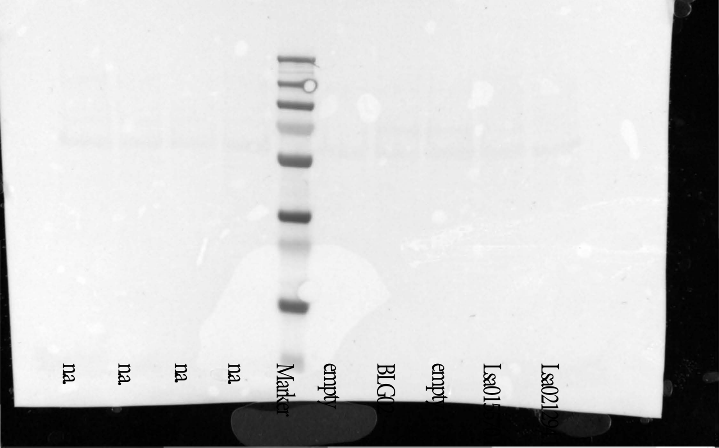

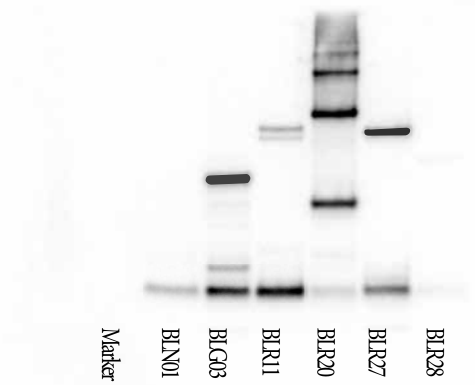

Chemiluminescence _blot _effector proteins2_detection 1 sec

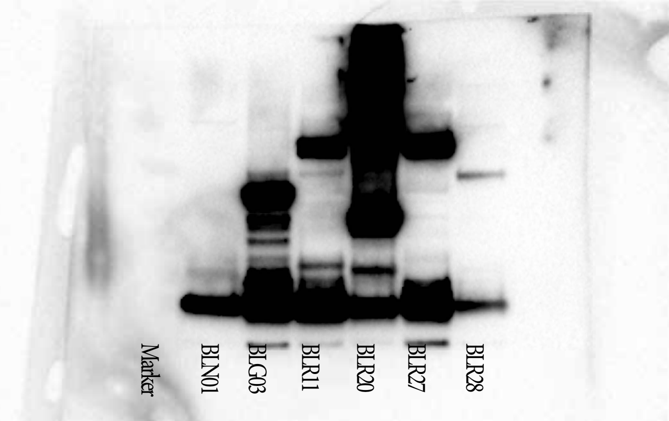

Chemiluminescence _blot _effector proteins2_detection 37 sec

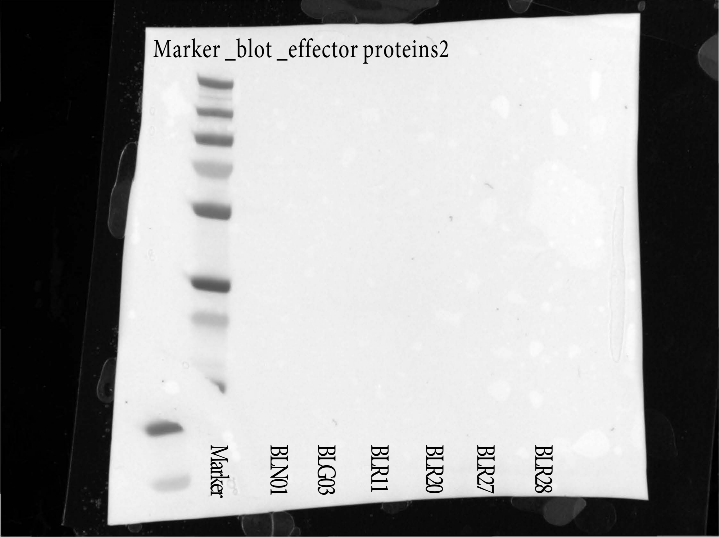
Marker _blot _effector proteins2
Marker
BLN01
BLG03
BLR11
BLR20
BLR27
BLR28

Chemiluminescence_blot_target_proteins3

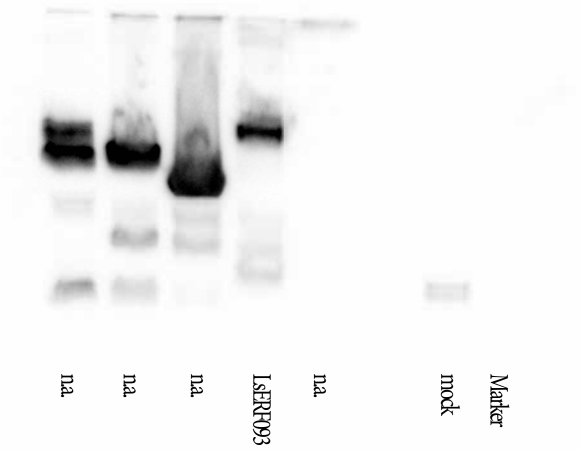

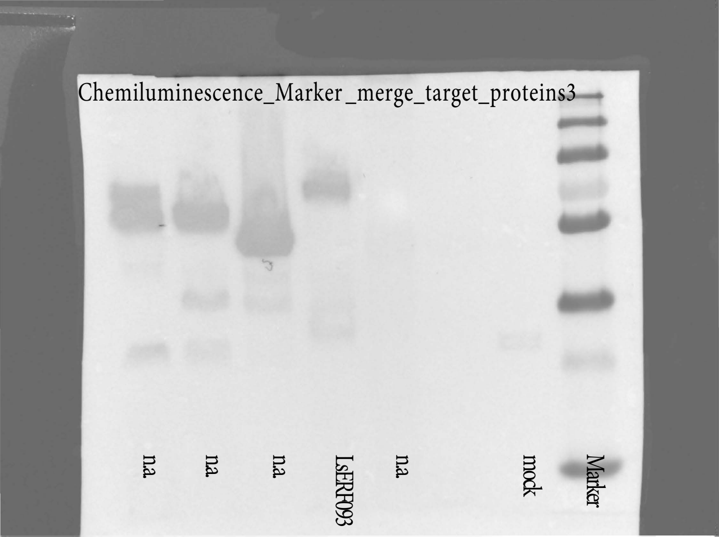
Chemiluminescence_Marker_merge_target_proteins3
n.a.
n.a.
n.a.
LsERF093
n.a.
mock
Marker
